# Health-related quality of life in children with chronic immune thrombocytopenia in China

**DOI:** 10.1186/s12955-016-0445-3

**Published:** 2016-03-15

**Authors:** Heng Zhang, Li Wang, Meijie Quan, Jie Huang, Peng Wu, Qin Lu, Yongjun Fang

**Affiliations:** Department of Hematology and Oncology, Nanjing Children’s Hospital Affiliated to Nanjing Medical University, Nanjing, China; Department of Hematology and Oncology, Children’s hospital of Hebei province, Shijiazhuang, China

**Keywords:** Immune thrombocytopenia (ITP), Health RELATED QUALITY OF life (HRQoL), The Kids’ITP Tools (KIT), The pediatric quality of life inventory™ (PedsQL™)

## Abstract

**Background:**

The concept of health-related quality of life (HRQoL) was brought up decades ago and has been well utilized in many different areas. Regarding immune thrombocytopenia (ITP) management, much work has been done to emphasize the necessity of taking HRQoL into consideration. However, data on HRQoL of children with chronic ITP remain rare.

**Methods:**

This is a cross-sectional study. Children with chronic ITP aged from 2 to 18 and their parents were recruited. Participants completed the Pediatric Quality of Life Inventory™ (PedsQL™) and Kids’ ITP Tools (KIT) questionnaires at only one time. The Pearson’s correlation was examined between these measures for the pooled samples.

**Results:**

A total of 42 families participated. Mean child self-report scores of KIT and PedsQL™ were 78.60 (SD = 12.40) and 85.13 (SD = 14.12), respectively, corresponding to parent proxy report scores, which were 73.40 (SD = 19.96) and 85.10 (SD = 13.56), respectively. Mean score of KIT parent impact report was only 40.39 (SD = 19.96). Significantly higher KIT scores (self-report and parent proxy) were observed in children with PLT more than 30 × 10*9/L compared to others, and this difference was even more noticeable in the PedsQL™ parent proxy report group (*p* < 0.001). As with intravenous immunoglobulin, the statistics difference appeared only in KIT child self-report group (*p* = 0.03), while for bone marrow examination, the difference appeared only in PedsQL™ parent proxy report group (*p* = 0.01). A negative relationship was apparent between duration of disease and scores. Gender and use of corticosteroids had no impact on the KIT and PedsQL™ scores here. Internal consistency reliability was demonstrated with Cronbach’s alpha for all scales above the acceptable level of 0.89 (range from 0.88 to 0.97). There was a substantial concordance (*p* < 0.001) between the child and parent proxy scores (ICC for KIT is 0.59, ICC for PedsQL™ is 0.85). Meanwhile, KIT scores are correlated with PedsQL™ (*r* = 0.75 for child self report, *r* = 0.61 for parent proxy report).

**Conclusions:**

ITP affects HRQoL of children and parents. Parents are much more concerned with the disease than their children, which seriously influence their HRQoL as a result. The cross-culture translated KIT is reliable and valid with acceptable correlation to the PedsQL™. KIT provides valuable information of childhood ITP and will be a reliable outcome measure for further clinical research on HRQoL.

## Background

Immune thrombocytopenia (ITP), characterized by acquired decrease in peripheral blood platelets and suppression of platelet production, is associated with some symptoms such as spontaneous bruising, mucosal bleeding, epistaxis, or to severe even fatal bleeding events and intracranial hemorrhage [1, 2]. Based on current estimation, the prevalence of ITP in children is about 4–6 cases per 100,000 annually [3]. Approximately, 20–25 % children with ITP become chronic (defined as peripheral blood platelet count <100 × 10*9/L and lasting for over 12 months) [4–9]. Recent reports show that for those diagnosed as chronic ITP, around 50 % children spontaneously recover within 5 years and a few may take more years [6]. ITP children’s families have to deal with incessant worries of bleeding, fears of invasive procedures (like blood tests, bone marrow aspiration and intravenous therapy) and risk of splenectomy, which will obviously affects their quality of life [10, 11]. Moreover, restrictions on lifestyle and the side effects of corticosteroid therapy, the most serious such as avascular necrosis, could even worsen it [10].

Recent guidelines emphasize the necessity of taking the issues on health related quality of life (HRQoL) into consideration while making decisions on management in childhood ITP, rather than just focusing on increasing platelet counts [12]. Some researches also support HRQoL to play a major role in treating patients with chronic diseases, which attracts our attention to explore the quality of life of childhood chronic ITP [13, 14]. HRQoL assessment provides a comprehensive vision of the patients’ subject feeling of well-being and function and, in the meantime, supports analyzing treatment effects [14]. The Kids’ ITP Tools (KIT), developed by Robert Klaassen and Barnard et al. from Canada, is a well-known disease-specific measure of HRQoL for childhood ITP patients and families [15, 16]. Till now, it has been validated in several countries, including UK, France, Germany, Uruguay and Egypt. However, China has only a few research records on chronic ITP HRQoL, which barely related to health improvement [17]. The Pediatric Quality of Life Inventory™ (PedsQL™) is a general measurement on HRQoL of children, which has been widely used around the world [18–20]. Thusly, we conduct a cross-section study to evaluate HRQoL in Chinese childhood chronic ITP patients by using KIT and PedsQL™. The objective of this prospective study is to improve the understanding of these patients and their families’ experience of life and advocate using QoL as key factor when considering clinical treatment.

## Methods

### Patients

From December 2013 to January 2015, 42 subjects with chronic ITP and their parents were recruited from the Nanjing Children’s Hospital Affiliated Nanjing Medical University and Children’s Hospital of Hebei Province. Parents or legal guardians of the subjects were interviewed in person to collect clinical information. The 42 subjects included 29 boys and 13 girls, with an median age of 5.79 (SD = 2.867). The lower limit of 2 years was used since the KIT was designed for children of 2 year-old or above. In the absence of a gold-standard test for ITP, the diagnosis of chronic ITP was based on isolated thrombocytopenia (platelet count < 100 g/L lasting for over 12 months) without any identified causes (normal hemoglobin level and white cell count and no hepatosplenomegaly or lymphadenopathy or significant abnormalities on blood smear, and no other chronic illness). Patients with secondary ITP associated with immune deficiency, collagen vascular disorder, or other causes were excluded. Children ≥7 years old were required to complete self-report versions of the KIT and self-report versions of the PedsQL™.

### Measures

This is a cross-sectional study with the questionnaires being completed by the participants at one time only. The generic and disease-specific HRQoL questionnaires were combined in the study. KIT is a disease-specific HRQoL measure that was initially developed and validated in North America and subsequently developed for use in other languages and cultures [15, 17]. KIT can be used in both clinic and home settings [15]. The measure consists of 3 questionnaires: one version for the child aged from 7 to 18 (child self-report), one for the parent to complete on behalf of the child aged from 2 to 18 (parent proxy report) and another for the parent to complete (parent impact report) [16]. The children’s version of the KIT consists of 25 items and parents’ version consists of 26 items. They were divided into separate domains as follows: children’s version: treatment side effects, intervention-related, disease-related, activity- related, and family-related concerns; parental version: diagnosis-related, monitoring-related, child’s restricted activity-related, daily life-related, disease outcome and emotional impact [16]. Scores for the individual domains are summed to yield a total KIT score. KIT scores have a range of 0 (worst) to 100 (best). Children ≥7 years old were asked to complete a self-report KIT, and parents completed a proxy KIT for their children and self-report for them.

PedsQL™ has undergone rigorous psychometric testing, and also has child and parent report components that are valid, reliable and responsive in the target countries [18–20]. The Chinese version of PedsQL™ has been in use regionally for at least 10 years and proven well established [21, 22]. We used the young child version (parent-assisted) for 5–7 years old, the child version (self-report) for 8–12 years old and the teen version (self-report) for 13–18 years old, and the 2–5, 5–7, 8–12 years parent-report versions [19, 20]. Scores for PedsQL™ also range from 0 (worst) to 100 (best).

The age-specific questionnaires were handed in hard copy to parents and patients in clinic and were filled out in the waiting room. Local investigators sent completed questionnaires by mail to the coordinating investigator. Data were entered in an electronic database by researchers of the coordinating study center.

Data checks and cleaning were performed on all data. All data were double entered to ensure accuracy. If 25 % or more of the item data were missing, the summary score would not be calculated.

### Statistical analysis

Statistical analyses here were performed using Statistical Package for Social Science (version 14.0; SPSS). Qualitative variables were described in number and percentages and mean ± SD, if quantitative. Internal consistency of the Chinese version of the KIT was analyzed by measuring Cronbach α test in the whole sample according to different respondents. Independent sample test was used to compare between means of different groups of patients. ANOVA tests were used when comparing quantitative data between more than 2 groups. The Pearson and Spearman coefficients were used when studying the relationship (direction and power) of quantitative variables simultaneously. Linear regression analysis was used to examine the extent to which a set of variables independently predicts a dependent variable. Two-sided P values were selected and *P* < 0.05 was considered statistically significant.

## Result

### Patients’ characteristics

The frequency distributions of all subjects of the selected variables are summarized in Table 1. The median age was 5.79 years old along with median age at diagnosis of ITP was 4.47 years old. Of the 42 chronic ITP patients, 37 children underwent bone marrow (BM) aspiration at diagnosis. The treatment protocol varied and included oral and intravenous steroids, immunoglobulin, and splenectomy and platelet infusion. Thirty-six children (85.7 %) received corticosteroids treatment. Twenty-five (59.5 %) received intravenous immunoglobulin (IVIG). Of all the patients, only one child received splenectomy and none had received platelet infusion. There are 16 patients with Platelet count (PLT) lower than 30 × 10*9/L at the past week of investigation. The diagnosis of chronic ITP is in accordance with classification of the American Society of Hematology 2011 evidence-based practice guideline for immune thrombocytopenia [12].

**Table 1:** Frequency distribution of selected variables in ITP patients

	Patients (*n* = 42)	
Variables	n	%
Median age (years)	5.79	
Median age at diagnosis of ITP	4.47	
Gender		
Male	29	69.0
Female	13	31.0
PLT (×10*9/L)		
≤30	16	38.1
> 30	26	61.9
BM	37	88.1
IVIG	25	59.5
Corticosteroids	36	85.7
Splenectomy	1	2.4
Platelet infusion	0	0

*PLT* Blood platelet, *IVIG* Intravenous immunoglobulin, *BM* Initial bone marrow examination

### PedsQL™ and KIT scores of children

The results of this comparison are shown in Table 2. All patients had completed the parent version of the KIT and PedsQL™ and for the child self-assessment version. Twenty-four patients were old enough to complete it independently.

**Table 2:** Scores for child/proxy groups and reliability of KIT and PedsQL™

HRQoL Scales	N	Scores			Reliability	
		Mean values (SD)	MINI	MAX	Cronbach’s alpha	ICC
KIT						
Child self-report	24	78.60 (12.40)	55.21	98.68	0.88	0.59
Parent proxy report	42	73.40 (19.96)	17.31	100.00	0.97	
PedsQL™						
Child self-report	22	85.13 (14.12)	63.04	100	0.89	0.85
Parent proxy report	40	85.10 (13.56)	55.68	100	0.89	

The mean score for KIT child self-report was 78.60 (SD = 12.40), ranging from 55.21 to 98.68. For parent proxy report and parent impact report measurement, the scores were 73.40 (SD = 19.96, range from 17.31 to 100) and 40.39 (SD = 19.96, range from 6.73 to 81.73), respectively. The PedsQL™ score was slightly higher with child-report mean of 85.13 (SD = 14.12; range from 63.04 to 100) and parent proxy report mean of 85.10 (SD = 13.56; range from 55.68 to 100). There were no significant differences between total scores of proxy and child self-report in PedsQL™ or KIT (*p* = 0.527 and *p* = 0.095, respectively). But it was observed that scores of children report were higher than those of parent proxy report in KIT, the difference approached but did not achieve statistical significance (*p* = 0.095) which did not differ in PedsQL™.

Interval between intervention and KIT was within 4 weeks. No major relation was found between gender and corticosteroids of the patients with either scores of child/proxy reports or parent reports. But it was observed that significantly higher KIT scores (self-report and parent proxy) in children with PLT more than 30 × 10*9/L compared to the group of PLT less than 30 × 10*9/L. This difference was reinforced in the PedsQL™ Parent report group (*p* < 0.001). As with intravenous immunoglobulin, the statistics difference appeared only in KIT child self-report group (*p* = 0.03), while for bone marrow examination, the difference appeared only in PedsQL™ parent proxy report group (*p* = 0.01).

Table 4 describes the correlations between mean scores and age at onset, as well as duration of disease and platelet count. As expected, a negative or inverse relation was apparent between duration of disease and scores. This was statistically significant in KIT Parent report Scores (*r* = −0.426, *p* = 0.005) and PedsQL™ child self report scores (*r* = −0.461, *p* = 0.031). A significantly positive correlation was found between platelet count and scores of KIT Child self-report, PedsQL™ Child self-report and PedsQL™ Parent report (*r* = 0.436, *p* = 0.033 and *r* = 0.531, *p* = 0.011 and *r* = 0.505 *p* = 0.001, respectively). Age at onset did not seem to be correlated with the KIT and PedsQL™ scores in patients.

### Validation and reliability

The assessment of validity was based on the Pearson’s correlation between the KIT and PedsQL™. The results showed the correlation between the KIT and PedsQL™ scores was 0.75 for child self-report and 0.61 for parent proxy report. All correlations were statistically significant (*p* < 0.0001). Child self-report had the higher correlations with parent proxy report in PedsQL™(*r* = 0.85) and a lower one in KIT (*r* = 0.51). Internal consistency reliability was demonstrated with Cronbach’s alpha for all scales above the acceptable level of 0.89 (range: 0.89–0.97). As Table 2 shows, there was also a substantial concordance (*p <* 0.001) between the children’s summary scores and the parent proxy report scores for the two measures (ICC for KIT is 0.59, ICC for PedsQL™ is 0.85).

## Discussion

This is the first study to identify HRQoL of childhood chronic ITP in China. We found that chronic ITP could have adverse effects on children’ and their parents’ functioning and well-beings, although some observations indicated that newly diagnosed childhood ITP had a significant impact on patients and their families [6]. Those parents and children usually have impaired HRQoL, as a result of the limitation of daily activity, disturbed sleep, social and emotional functioning. Known as a self-limiting disease, childhood ITP leads to a very low rate of mortality and severe morbidity [10, 23]. But confronted with chronic ITP, the physicians do not know whether and when will the patients recover, which brings endless anxiety to the patients and the families. When faced with unpredictable bleeding events as well as the adverse effects of treatment, children and the families show more concerns, therefore affects the whole families’ quality of life. To better fit the subject’s perspectives and help develop a more efficient clinic therapy, both KIT and PedsQL™ questionnaires were used here to systemically analyze HRQoL in children with chronic ITP. The study demonstrates that the KIT remains valid and reliable and adaptable to Chinese culture after translation into Chinese.

The median age of patients was 5.79 years old, while the median age at diagnosis was 4.47 years old, which agreed with the common stating that ITP most commonly occurred in children from 4 to 6 [12, 24]. Scores of parent proxy report stay lower than child self-report group of KIT, however, scores of PedsQL™ did not make a difference. The reason for these findings could be that the PedsQL™ only focus on the general parts such as the ability to walk, run and so on, which may not be restricted in most children, while KIT pays more attention to the disease-related function. This difference may reflect parents’ concerns are more about disease-related impact, while children adapt to their situation faster than their parents. This discrepancy was comparable to Zilber’s study, which has been proven a common finding by lots of researches [25]. As Rama Zilber mentioned, the basic concern of all parents was about the health and future of their children, the life experiences and inner life of children were different from adults, which reflected their different priorities [25].

Many consensuses have reported that a BM aspirate should be performed in children with newly diagnosed ITP and atypical features [12, 26, 27]. Some researchers believe that conducting a BM test become a major issue bothering children. In this study, as with bone marrow examination, the statistics difference appeared only in PedsQL™ parent proxy report group (*p* = 0.01), there is no significant difference in child self-report parts in both questionnaires. As shown in Table 3, scores of ever taking invasive procedures was much higher than others in PedsQL™ Parent report group. In China, BM examination is used as necessary diagnosis tools to exclude other causes of thrombocytopenia. Diagnosed with ITP may contribute to HRQL of the parents, as a consequence of reduce in concerns about other malignant diseases may have occurred to their children.

**Table 3:** Comparison between scores of child/proxy and parent self-reports in relation to clinical characteristics and treatment of ITP

Clinical Characteristics and Treatment of ITP		KIT				KIT				KIT				PedsQL™				PedsQL™			
		Child self-report				Parent proxy report				Parent impact report				Child self-report				Parent proxy report			
		N	Mean	SD	*P* ^a^	N	Mean	SD	*P* ^a^	N	Mean	SD	*P* ^a^	N	Mean	SD	*P* ^a^	N	Mean	SD	*P* ^a^
Gender	Male	19	79.29	12.39	0.64	29	72.48	18.92	0.66	29	42.40	17.57	0.29	18	85.40	14.30	0.70	28	85.69	14.25	0.83
	Female	5	76.02	13.48		13	75.46	22.78		13	35.92	17.75		4	84.37	12.22		12	82.61	15.37	
Platelet count	≤30	8	69.80	14.57	**0.01**	16	63.45	21.05	**0.01**	16	34.99	19.35	0.11	8	78.57	14.44	0.14	17	75.44	14.45	**0.00**
	>30	16	83.01	8.63		26	80.17	16.35		26	44.06	15.78		14	88.19	13.36		23	91.53	8.23	
BM	Never	3	82.51	7.70	0.57	5	65.48	29.57	0.35	5	45.41	20.99	0.51	1	63.04	0.00	-	3	67.06	6.87	**0.01**
	Ever	21	78.05	12.97		37	74.47	18.62		37	39.71	17.39		21	86.18	13.56		37	86.56	12.94	
IVIG	Never^c^	9	84.45	7.00	**0.03**	17	70.85	17.81	0.50	17	37.05	21.19	0.32	8	86.14	13.94	0.80	16	81.53	11.72	0.17
	Ever^b^	15	75.10	13.76		25	75.14	21.48		25	42.66	14.85		14	84.55	14.72		24	87.47	14.41	
Corticosteroids	Never^c^	3	81.22	8.11	0.70	6	63.51	28.86	0.19	6	38.29	28.62	0.85	2	81.52	26.13	0.71	5	82.09	11.73	0.60
	Ever^b^	21	78.23	13.00		36	75.05	18.11		36	40.74	15.74		20	85.49	13.53		35	85.52	13.91	

Value in bold indicate significant *P* value

*P* ≤ 0.05: significant

*P* ≤ 0.01: highly significant

*IVIG*, Intravenous immunoglobulin, *PLT,* Platelet count, *BM*, Initial bone marrow examination

^a^Tested by ANOVA test

^b^Ever mean ever taking intervention within 4 weeks of questionnaires

^c^Never mean never taking intervention within 4 weeks of questionnaires

As can be told from Tables 3 and 4, in parent impact report and child self-report and proxy report group, PLT, was proportionally correlated with HRQoL scores. The obvious low scores from parents indicate their much more concerns on PLT issue. This result contrasted to the report of Neunert et al. [10]. Although some guidelines declared patients with absent or minor bleeding should not be treated regardless of platelet count, parents were still bothered by the low PLT or the fear of unpredictable bleeding episodes. Most parents in China pay much attention to avoid bleeding events, as a result, much of the daily activities were limited and might reduce the children’s quality of life. Education, reassurance and management plan will reduce the worry and improves quality of life.

**Table 4:** Correlations between scores of two measures and ITP clinical characteristics

	KIT		KIT		KIT		PedsQL™		PedsQL™	
	Child self-report		Parent proxy report		Parent self-report		Child self-report		Parent proxy report	
	*r*	*P*	*r*	*P*	*r*	*P*	*r*	*P*	*r*	*P*
Age at onset (year)^a^	0.130	0.546	−0.064	0.689	0.199	0.206	0.191	0.395	−0.038	0.814
Duration (month)^a^	−0.060	0.782	**−0.426** ^**b**^	**0.005** ^**b**^	0.243	0.121	**−0.461** ^**c**^	**0.031** ^**c**^	−0.150	0.354
Platelet count	**0.436** ^**c**^	**0.033** ^**c**^	0.286	0.067	0.231	0.141	**0.531** ^**c**^	**0.011** ^**c**^	**0.505** ^**b**^	**0.001** ^**b**^

Value in bold indicate significant *P* value

^a^Correlated by the Spearman correlation

^b^Correlation is significant at the 0.01 level (2-tailed)

^c^Correlation is significant at the 0.05 level (2-tailed)

Substantial side effects associated with chronic ITP have been reported recently. The treatment of chronic ITP in China is in accordance with the American Society of Hematology 2011 evidence-based practice guideline for immune thrombocytopenia: 1) Children with no bleeding or mild bleeding (defined as skin manifestations only, such as bruising and petechiae) be managed with observation alone regardless of platelet count. 2) For pediatric patients requiring treatment, a short course of corticosteroids or a single dose of IVIG (0.8–1 g/kg for 1 or 2 days, or 0.4 g for 3–5 days) be used as first-line treatment. 3) A single dose of anti-D can be used as first-line treatment in Rh-positive, nonsplenectomized children requiring treatment. 4) High-dose dexamethasone may be considered for children or adolescents with ITP who have significant ongoing bleeding despite treatment with IVIG, anti-D, or conventional doses of corticosteroids. 5) Splenectomy or other interventions with potentially serious complications are the last choices which should be delayed for at least 12 months [12]. In subject design phase, we supposed that treatment with corticosteroids and intravenous immunoglobulin could improve the HRQoL. We presume that the treatment would increases platelet count observed in the majority of patients and provide a concurrent lower risk of bleeding, reducing the fear of bleeding events. This association has not been well verified with corticosteroids here. As with intravenous immunoglobulin, the significant difference appeared only in KIT Child self-report group. For corticosteroids, the adverse effects most often reported by patients were moon face, weight gain, personality changes, psychosis, diabetes, cataract and osteopenia which could decrease HRQoL of patients [11, 28]. As for intravenous immunoglobulin, the adverse effects (high donor exposure, risk of transmitted infection, fever, hemolyis, headache, asceptic meningitis) and require of intravenous cannula, stay in hospital to get infusion of immunoglobulin may reduce the overall score. While balancing pros and cons in intravenous immunoglobulin, children obviously consider more of the disadvantages rather than the benefits. The impact of adverse effects may counterbalance a potentially positive effect of corticosteroids on HRQoL scores. This is partly concordant with the results of Heitink-Polle et al. and Grainger et al., which showed HRQoL was not improved by treatment [4, 29].

No significant relation was found between age at onset or gender with either scores of child/proxy reports or parent reports. Strullu et al. reported no significant association had been found between sex, age and PedsQL™ scores as well [30]. The effect of splenectomy as a treatment option was not addressed in the study since only 1 respondent had that.

Some limitations of the study should be addressed. Firstly, the study was limited by the small sample size of subjects. The age of children should be more than 7 to finish the child self-report part independently, while the median age of ITP children here is 5.79 years old, which severely reduces the number of qualified subjects. It is essential to validate the results in a larger size of subjects. Secondly, focus was only placed on the traditional treatment such as IVIG and corticosteroids rather than some new treatment. Parents in China prefer taking traditional Chinese medicines concurrently. Many traditional Chinese medicines contain steroid extracts, which are not taken into discussion in this report. Future research should take limitations above into account.

ITP is generally a benign disease, while the specificity of chronic diseases has a strong impact on HRQoL of patients and their families. The research shows that HRQoL of ITP children and their parents were influenced by duration of the disease, the PLT, BM and intravenous immunoglobulin. Physicians should treat chronic ITP children with awareness of the families’ anxieties and closely follow up side effect of invasive procedures and treatments. KIT can succinctly summarize the patient and family’s perspective of HRQoL in a more understandable manner to clinicians. It will be a reliable outcome measure for further clinical research of HRQoL of childhood ITP.

## Conclusions

ITP affects HRQoL of children and parents. Parents are much more concerned with the disease than their children, which seriously influence their HRQoL as a result. The cross-culture translated KIT is reliable and valid with acceptable conrrelation to the PedsQL^TM^. KIT provides valuable information of childhood ITP and will be a reliable outcome measure for further clinical research on HRQoL. 

## Ethics approval and consent to participate

The research protocol was approved by the Medical Ethics Committee of Nanjing Children’s Hospital affiliated to Nanjing Medical University. Written informed consent was obtained from the parents or legal guardians of all study subjects at the time of enrollment into this study.

## Abbreviations

HRQoL: health-related quality of life
ITP: immune thrombocytopenia
IVIG: intravenous immunoglobulin
KIT: Kids’ ITP tools
PedsQL™: pediatric quality of life inventory™
PLT: platelet count
SPSS: statistical package for social science
